# Human Metaplastic Breast Carcinoma and Decorin

**DOI:** 10.1007/s12307-017-0195-8

**Published:** 2017-06-26

**Authors:** Pia Boström, Annele Sainio, Natalja Eigėlienė, Anne Jokilammi, Klaus Elenius, Ilkka Koskivuo, Hannu Järveläinen

**Affiliations:** 1 Department of Pathology, University of Turku and Turku University Hospital, Kiinamyllynkatu 10, 20520 Turku, Finland; 2 Department of Medical Biochemistry and Genetics, University of Turku, Kiinamyllynkatu 10, 20520 Turku, Finland; 3 Department of Cell Biology and Anatomy, University of Turku, Turku, Finland; 4 Oncology Clinic, Vaasa Central Hospital, Vaasa, Hietalahdenkatu 2-4, 65130 Vaasa, Finland; 5 Department of Oncology and Radiotherapy, University of Turku and Turku University Hospital, Turku, Finland; 6 Department of Plastic and General Surgery, Turku University Hospital, Kiinamyllynkatu 4-8, 20520 Turku, Finland; 7 Department of Internal Medicine, Satakunta Central Hospital, Sairaalantie 3, 28500 Pori, Finland

**Keywords:** Adenoviral transduction, Decorin, Metaplastic breast carcinoma, Three-dimensional (3D) explant culture

## Abstract

Metaplastic breast carcinoma (MBC) is a rare subtype of invasive breast cancer and has poor prognosis. In general, cancers are heterogeneous cellular masses comprised of different cell types and their extracellular matrix (ECM). However, little is known about the composition of the ECM and its constituents in MBC. Decorin is a ubiquitous ECM macromolecule known of its oncosuppressive activity. As such, it provides an intriguing molecule in the development of novel therapeutics for different malignancies such as MBC. In this study, decorin immunoreactivity and the effect of adenoviral decorin cDNA (Ad-DCN) transduction were examined in MBC. Multiple immunohistochemical stainings were used to characterize a massive breast tumour derived from an old woman. Furthermore, three-dimensional (3D) explant cultures derived from the tumour were transduced with Ad-DCN to study the effect of the transduction on the explants. The MBC tumour was shown to be completely negative for decorin immunoreactivity demonstrating that the malignant cells were not able to synthesize decorin. Ad-DCN transduction resulted in a markedly altered cytological phenotype of MBC explants by decreasing the amount of atypical cells and by inhibiting cell proliferation. The results of this study support approaches to develop new, decorin-based adjuvant therapies for MBC.

**Electronic supplementary material:**

The online version of this article (doi:10.1007/s12307-017-0195-8) contains supplementary material, which is available to authorized users.

## Introduction

Metaplastic breast carcinoma (MBC) is a rare subtype of invasive breast cancer accounting for 0.2–5% of all breast malignancies [1]. MBC is a descriptive term and comprises histologically a broad range of invasive breast tumours characterized by co-existence of two or more cellular types. Commonly MBC consists of epithelial (carcinomatous) and mesenchymal (sarcomatous) elements in a variable proportion [2]. MBC is regarded as ductal carcinoma that undergoes metaplasia into squamous cell, spindle cell and/or mesenchymal appearance [3]. Multiple immunohistochemical stainings are required for accurate diagnosis of these tumours because of their various histological patterns. Immunohistochemical analyses have shown that >90% of MBCs are negative for estrogen receptor (ER), progesterone receptor (PR) and human epidermal growth factor receptor 2 (HER2/neu), and that they have a consistent expression of basal markers such as cytokeratin 5/6 (CK 5/6) and/or epidermal growth factor receptor (EGFR) [4]. Thus, most MBCs are regarded as a subtype of triple negative breast cancer (ER, PR and HER2/neu negative). For women presenting with triple negative breast cancer, hormonal therapy is generally unsuccessful, albeit initial response to chemotherapy can be good [5]. MBC has also a poor clinicopathological response to local radiotherapy [6]. Subsequently, 5-year overall survival rate of patients with MBC is only 54,5% [7].

MBC is usually associated with a lower incidence of axillary nodal involvement compared to typical breast carcinomas of similar size [8]. Nevertheless, MBC tends to have high percentage of local recurrence and distant metastases, particularly in the lung [9]. The different histological subtypes of MBC are associated with distinct clinical behaviour and outcome [10]. Spindle cell and squamous cell subtypes have been shown to associate with a particularly aggressive behaviour [11]. Studies have also shown that MBC may be enriched by primitive cells with stem-like features. This may account for the resistance of the tumour to chemotherapy and enhanced metastatic potential [12, 13]. Thus, new and novel adjuvant therapies are needed.

In general, cancers are heterogeneous cellular masses comprised of various cell types including malignant cells, immune cells, epithelial cells, stromal cells and their extracellular matrix (ECM) [14, 15]. Cells in the tumour microenvironment (TME) can be harnessed by malignant cells to possess tumour-promoting function at all stages of tumourigenesis [16–18]. On the other hand, ECM proteins expressed by various stromal cell types can regulate the behaviour of cancer cells and thus affect both cancer progression and metastasis [19–21]. This offers possibilities to target malignant cells, mediators of their communication or macromolecules in the TME to improve the therapeutic prognosis of the disease and to complement other treatment options [22–26].

Certain ECM macromolecules, particularly proteoglycans (PGs) such as decorin, have variously been identified as one of the key players capable of modulating cell signaling, adhesion, migration, proliferation, and apoptosis [27, 28]. Decorin is the prototype member of the small leucine rich proteoglycan (SLRP) gene family [29]. It is capable of interacting with various ECM macromolecules, receptors and growth factors, such as the members of the ErbB family [30–33]. Indeed, decorin is considered as a central modulatory molecule in various types of cancer [34, 35]. Normally, it is expressed by cells of mesenchymal origin such as fibroblasts and vascular smooth muscle cells [36, 37]. In cancers, including breast cancer, the expression of decorin in the TME has been reported to be markedly decreased or totally lacking in malignant cells [38–41]. Reduced level of decorin expression has been shown to be associated with poorer outcome in invasive breast cancer [42], and vice versa, high stromal decorin expression has been indicated to predict better prognosis [43]. Furthermore, decorin adenoviral transduction has been demonstrated to decrease the malignant behaviour of breast cancer cells [40] and even to suppress their capability to form bone metastasis [44, 45]. As a summary, decorin is currently considered as an oncosuppressive molecule [32, 46, 47]. Interestingly, its close structural relative, biglycan, has been shown to possess opposite function, namely tumour-promoting activity [48–51].

In this case report, a massive breast tumour was first characterized as MBC. Next, the MBC tumour was examined for immunoreactivity for the above two stromal PGs, decorin and biglycan. Thereafter, 3D MBC explant cultures were established and the effect of Ad-DCN transduction on the cytology and proliferation index of the explants was examined. Finally, the effect of Ad-DCN transduction on the expression of the ErbB family members, EGFR (ErbB1), ErbB2 (HER-2, c-Neu), ErbB3 and ErbB4 was also analyzed.

## Materials and Methods

### Case Presentation

We present a case of an 87 years old Finnish woman, who was admitted to Turku University Hospital, Turku, Finland, with a locally advanced breast cancer with ulceration. She was in moderate condition for her years. Her breast examination revealed breast tissue that was red, swollen and had a 3–4 cm ulceration with cystic cavity. The palpable tumour was over 10 cm in diameter. The study protocol was approved by the Joint Ethics Committee of the University of Turku and Turku University Hospital, Turku, Finland (number TO6/029/15). Informed consent was obtained from the patient.

### Immunohistochemistry

After radical mastectomy, a representative sample from the fresh breast tumour tissue (BTT) was obtained within half an hour of surgery. The tissue sample was taken from the invasive border of the tumour by a pathologist. Thereafter, the tumour tissue was divided into samples for the immunohistochemistry (IHC) analyses, and samples for the 3D transduction experiments.

The BTT samples for hematoxylin and eosin (HE) staining and IHC were fixed in 10% neutral-buffered formalin, embedded in paraffin and cut into 4 μm consecutive sections. The sections were stained with antibodies against ER (clone SP1; rabbit), PR (clone 1E2; rabbit), HER2 (clone 4B5; rabbit), EGFR (clone 5B7; rabbit), cytokeratin Pan (CkPan, clone AE1/AE3&PCK26; mouse), cytokeratin 7 (clone SP52; rabbit), p63 (clone 4A4; mouse), CD10 (clone SP67; rabbit), vimentin (clone V9; mouse), epithelial membrane antigen (EMA, clone E29; mouse), CK5/6 (clone D5/16B4; mouse) and Ki-67 (clone 30–9; rabbit) using Ventana Medical Systems/Roche Diagnostics with BenchMark XT immunostainer and *ultra*VIEW Universal DAB Detection Kit (Ventana/Roche; Tucson, Arizona, USA). All the above listed antibodies were ready-to-use dilutions (Diluent 95,119, Ventana/Roche). Antibodies against androgen receptor (AR, clone AR27; dilution 1:10; rabbit) and gross cystic disease fluid protein-15 (GCDFP-15, clone 23A3; dilution 1:10, rabbit) from Novocastra (Leica Biosystems; Newcastle, UK) were also used. The antibodies for AR and GCDFP-15 were diluted in Antibody Diluent from Ventana (251–018, Ventana/Roche).

Additionally, tumour sections were examined for immunoreactivity for decorin (DCN) and biglycan (BGN) [52]. Briefly, incubation with the primary decorin polyclonal rabbit antibody H-80 (dilution 1:50 in blocking buffer) and biglycan polyclonal goat antibody L-15 (dilution 1:50 in blocking buffer) from Santa Cruz Biotechnology (Heidelberg, Germany) were performed overnight at +4 °C while control sections were incubated in the blocking buffer, containing 2% bovine serum albumine (BSA) in phosphate-buffered saline (PBS). Next day, the sections were washed with PBS and incubated for 1 h with biotinylated secondary antibodies (dilution 1:200 in blocking buffer, Vector Laboratories, Inc., Burlingame). After rinses with PBS, the sections were treated with avidin-peroxidase complex solution (Vector Laboratories) for 35 min. Visualization of the signals was achieved with 3,3′ diaminobenzidine (Vector Laboratories), and the sections were counter-stained with Papanicolaou hematoxylin and mounted using Aquamount (BDH Laboratory Supplies, Dorset, England). List of all the primary antibodies used in the IHC stainings of the BTT sections can be seen as Table 1 in the Online Resource 1.

### 3D Explant Cultures

**Fig. 1:**
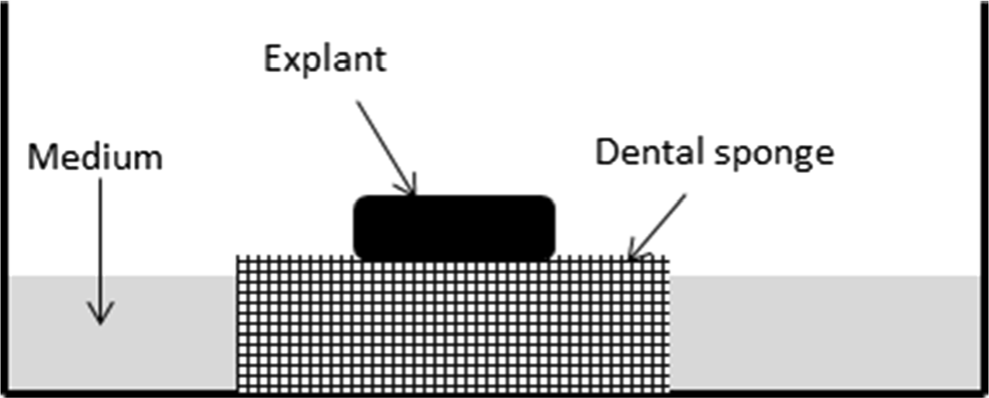
Schematic illustration of the in vitro three-dimensional explant culture

The BTT samples were transported to the cell culture laboratory in cold (+4 °C), sterile 0.9% sodium chloride (NaCl) supplemented with penicillin (100 IU/mL), streptomycin (100 μg/mL) and Fungizone (0,25 μg/ml). The 3D explant cultures were established as previously described with some minor modifications [53–55]. Briefly, the tumour tissue was cut with surgical scalpel in tissue pieces of approximately 2 × 2 × 2 mm and the pieces were rinsed few times in Dulbecco’s modified Eagle’s medium/nutrient mixture F-12 (DMEM/F12) without phenol red (GIBCO, Paisley, UK), supplemented with penicillin (100 IU/ml), streptomycin (100 μg/ml) and Fungizone (0,25 μg/ml). Thereafter, the tissue samples were transferred onto wet dental sponges (Spongostan™, Dental, Johnson & Johnson) in 12-well plates and cultured for 24 h in phenol-red-free DMEM/F12 supplemented with 10% fetal bovine serum (FBS), penicillin (100 IU/mL), streptomycin (100 μg/mL), Fungizone (0,25 μg/ml), 0.5 mL insulin-transferrin-selenium (ITS supplement, Sigma, Germany) and 100 nM hydrocortisone (Sigma). Schematic illustration of the 3D explant culture system is shown in Fig. 1. The explants were kept in a humidified atmosphere with a mixture of 5% CO_2_ and 95% air at +37 °C. After 24 h, the tumour explants were transduced as described below.

### Human Decorin cDNA Adenoviral Vector Transduction

The 3D MBC explant cultures were transduced with a recombinant replication-deficient adenoviral vector carrying human decorin cDNA (Ad-DCN) as previously described [40] with minor modifications. 24 h after the 3D explant cultures were established, the medium in the wells was substituted with fresh one and the explants were transduced with 100 plaque forming units (pfu)/cell of Ad-DCN or control vector carrying human LacZ gene (Ad-LacZ). Tissue sections without transduction were used as negative controls. The viral vectors were pipetted on top of the tumour explants in a droplet of the culture medium. The amount of the viruses was estimated using established tissue size 2 × 2 × 2 mm and the mean breast carcinoma cell size [56] resulting in 2,5 × 10^4^ cells in horizontal surface of the tissue section. In negative control cultures the medium was substituted with fresh one. After 24 h, the medium in the wells was replaced with fresh one. Next day, tissue explants and dental sponges underneath them were fixed in 4% paraformaldehyde for 48 h at +4 °C and embedded in paraffin. The paraffin blocks were cut into 4 μm consecutive sections and stained for HE and Ki-67 to evaluate the effect of Ad-DCN transduction on the cytology and the proliferation index of the explants.

### Visualization of HE and IHC, and Quantification of Ki-67

All tissue sections were scanned with Pannoramic Digital Slide scanner (The Pannoramic 250 Flash, 3DHISTECH Ltd., Hungary). Digital images were viewed with Pannoramic Viewer (3DHISTECH). Nuclear staining <1% of the tumour cells was considered negative for ER and PR. HER2 expression was evaluated as membrane staining of invasive tumour cells according to American Society of Clinical Oncology/College of American Pathologists (ASCO/CAP) guidelines [57]. CK5/6 and EGFR with membranous and/or cytoplasmic staining were considered positive if at least 10% of the cancer cells showed staining. p63 and AR immunoreactions presented with strong nuclear staining. CD10 stained the cell membranes and the cytoplasm of the tumour cells. CK7, CkPan, GCDFP-15, EMA and vimentin stained only the cytoplasm of the tumour cells. For the quantification of Ki-67 staining, 6 snapshots were taken from all stained samples and the quantification was performed with freely available ImmunoRatio software (http://153.1.200.58:8080/immunoratio/). The program automatically calculates the ratio of Ki-67 positive cells to total cell count.

### RNA Isolation and cDNA Synthesis

Total RNA was extracted from the 3D MBC explant cultures using TRIsure RNA isolation reagent (Bioline, London, UK). To eliminate possible contaminating DNA, RNA samples were treated with 10 units of DNase I (Roche). cDNA was synthesized in a reaction using 1 μg of total RNA and Sensifast cDNA Synthesis Kit (Bioline) according to the manufacturer’s protocol.

### Quantitative Real-Time PCR

Real-Time PCR (RT-qPCR) analyses of EGFR, ErbB2, ErbB3 and ErbB4 (JM-a) isoform expression was performed as previously described [58]. Expression of GAPDH was analysed as an internal control using primers 5′-AGCCACATCGCTCAGACAC-3′, 5′-GCCCAATACGACCAAATCC-3′ (Eurofins Genomics) and universal fluorescent probe #60 (Universal ProbeLibrary, Roche). Thermal cycling was performed with QuantStudio 12 K Flex Real-Time PCR System (Thermo Fisher Scientific, MA, USA).

### Statistical Analysis

The effect of Ad-DCN transduction on the proliferation index of the tissue explants was evaluated using Tukey’s Multiple Comparison Test. The *p* values <0.05 were considered statistically significant.

## Results

### Pathological Findings

**Fig. 2:**
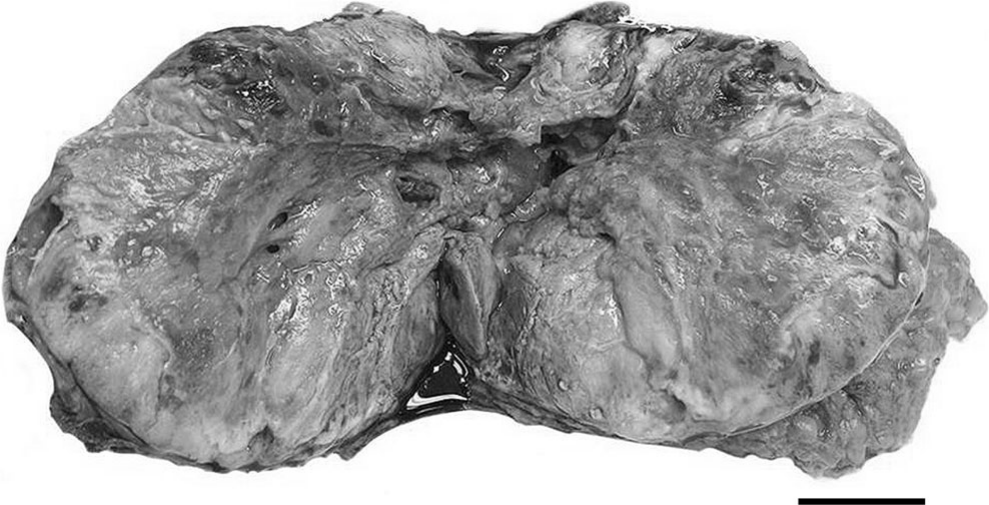
Cut section of the massive necrotic breast tumour. *Scale bar* 5 cm

Gross examination of the mastectomy specimen revealed a necrotic, cystic and haemorrhagic tumour mass measuring over 10 cm in diameter with poorly circumscribed infiltrative margins (Fig. 2). There was an extensive cutaneous ulceration with underlying dermal involvement by the tumour, but no fixation to the chest wall. The shortest surgical margin width was over 7 mm. No lymphovascular invasion was observed during operation. However, postoperative pathological results revealed metastases in 2 lymph nodes out of 29 lymph nodes in the right axilla. The largest lymph node metastasis was 12 mm in diameter. The general condition of the patient after the operation was satisfying. No further treatment after the surgery was planned because of her condition and old age. The patient died 6 months after the operation.

### Characterization of the Tumour

The histopathology showed that the tumour was composed of intersection bundles of pleomorphic spindle shaped cells with large pleomorphic hyperchromatic nucleus and moderate amount of cytoplasm. There were also round to polygonal cells arranged in irregular sheets and occasional ductal structures. Apocrine differentiation was found focally with various clusters of papillary projections and sheets. In addition, a small, irregular lace-like osteoid was seen. Mitotic figures were numerous (data not shown).

**Fig. 3:**
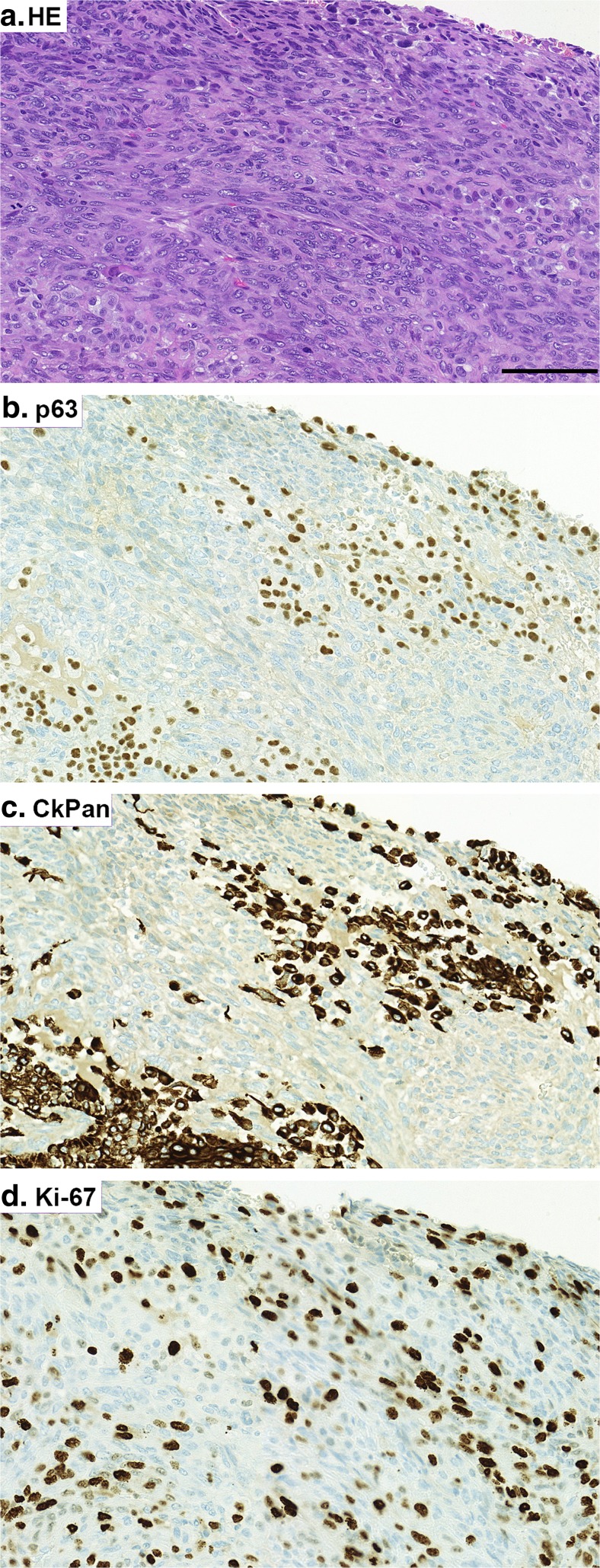
Representative images of selected immunohistochemical stainings of the massive breast tumour. HE staining (**a**). Immunohistochemistry (IHC) for p63 (**b**). IHC for CkPan (**c**). IHC for Ki-67 (**d**). Positive immunoreactivity can be seen in brown. Scale bar 100 μm. Note that there are areas within the tumour showing immunoreactivity for both CkPan and p63 (3**b** and **c**). This can be a display of carcinomatous epithelium transforming into metaplastic component

The immunohistological profile of the tumour revealed a neoplasm with a highly heterogeneous histology. Selected IHC images are shown in Fig. 3. The epithelial (carcinomatous) component that showed focal and patchy configuration, stained positive for CkPan and CK7. Moderate cytoplasmic or membranous staining of 70% of epithelial component was positive for CK5/6. The epithelial component was accounted less than 1–2% of the whole breast area. Mesenchymal component stained positive for vimentin and p63. Interestingly, some small areas of the tumour cells showed similar immunoreactivity for both CkPan and p63 (Fig. 3b and c). All tumour cells were ER, PR and HER2 negative. Ki-67 staining was positive in approximately 35% of the tumour cells. EGFR overexpression was assessed based on membrane staining, which showed strong intensity widely in the tumour area. In addition, CD10 positive cells were widely seen. There was also a sub-population of cells demonstrating apocrine differentiation by expressing AR, GCDFP-15 and EMA.

### Detection of Decorin and Biglycan in MBC Tissue

**Fig. 4:**
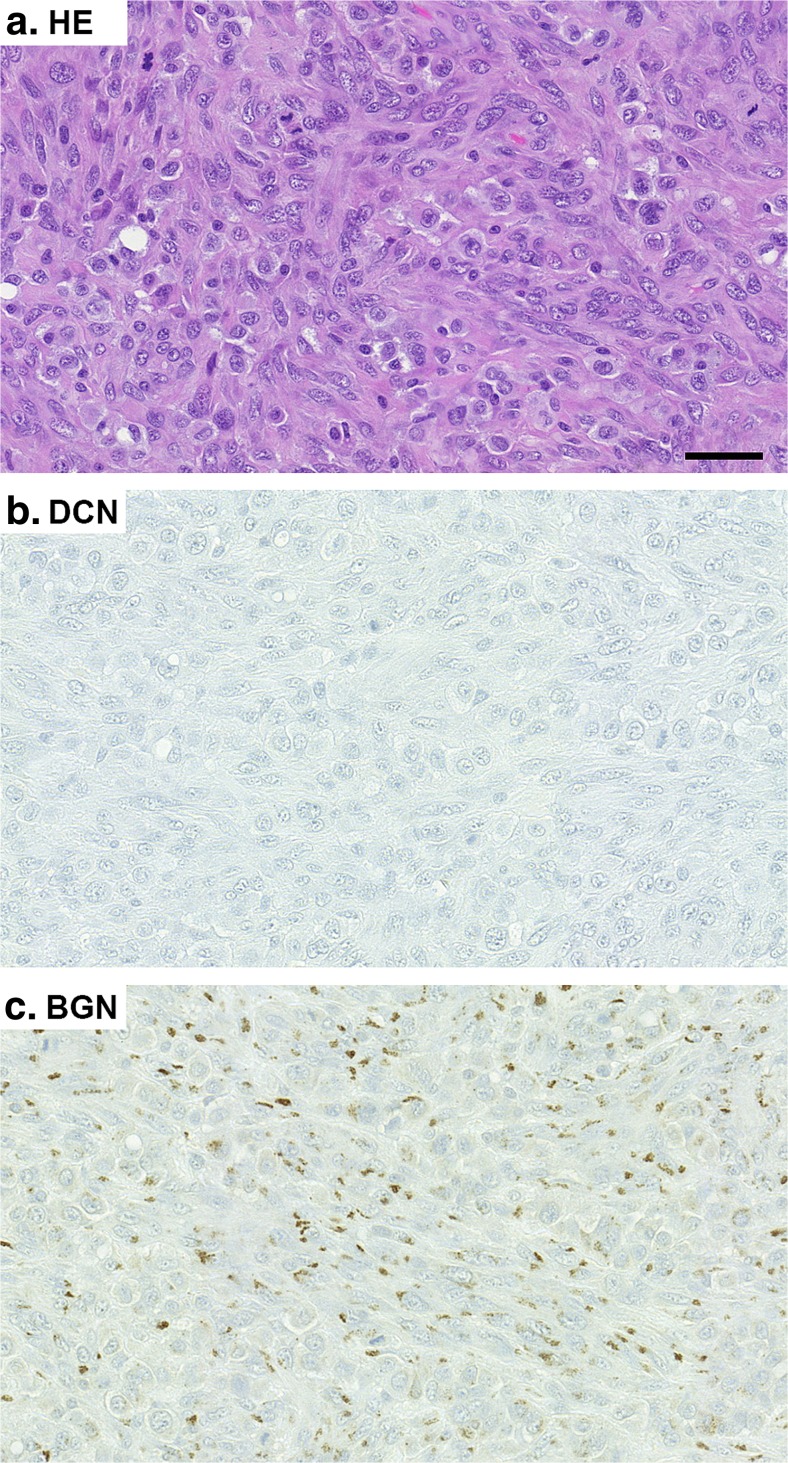
Immunohistochemistry (IHC) for decorin (DCN) and biglycan (BGN) in MBC tissue. HE staining (**a**). IHC for DCN (**b**). IHC for BGN (**c**). Positive immunoreactivity can be seen in *brown*. *Scale bar* 50 μm. Note that the MBC tumour is completely negative for DCN immunoreactivity while immunoreactivity for BGN is clearly detected

Next we examined the immunoreactivity for two stromal PGs, namely DCN and BGN, in this MBC tumour. The results demonstrated that the tumour tissue was completely negative for DCN immunoreactivity, while BGN immunoreactivity was locally clearly detected (Fig. 4).

### Effects of Human Decorin cDNA Transduction on 3D MBC Explant Cultures

**Fig. 5:**
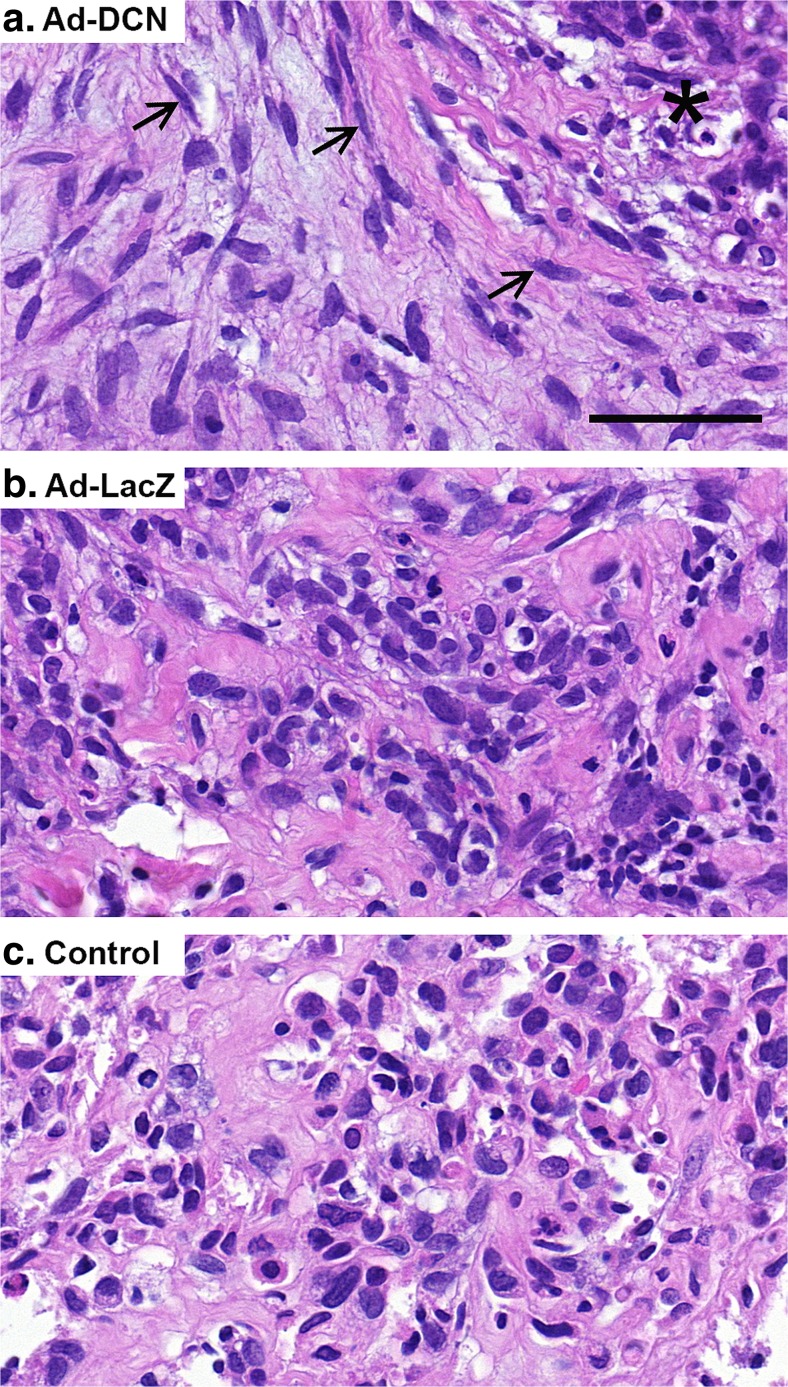
Representative HE images of MBC explant cultures after adenoviral transductions. Human decorin cDNA adenoviral transduction (Ad-DCN) (**a**). Adenoviral LacZ (vector control) transduction (Ad-LacZ) (**b**). Negative control explant (no viral transduction) (**c**). Note that Ad-DCN transduced explant cultures contain fibroblast-like cells (*arrows*) in a more organized manner compared to Ad-LacZ transduced explant cultures or to control cultures. *Asterisk* in image **a** indicates a representative area with cell debris and residual tumour cells. *Scale bar* 50 μm

**Fig. 6:**
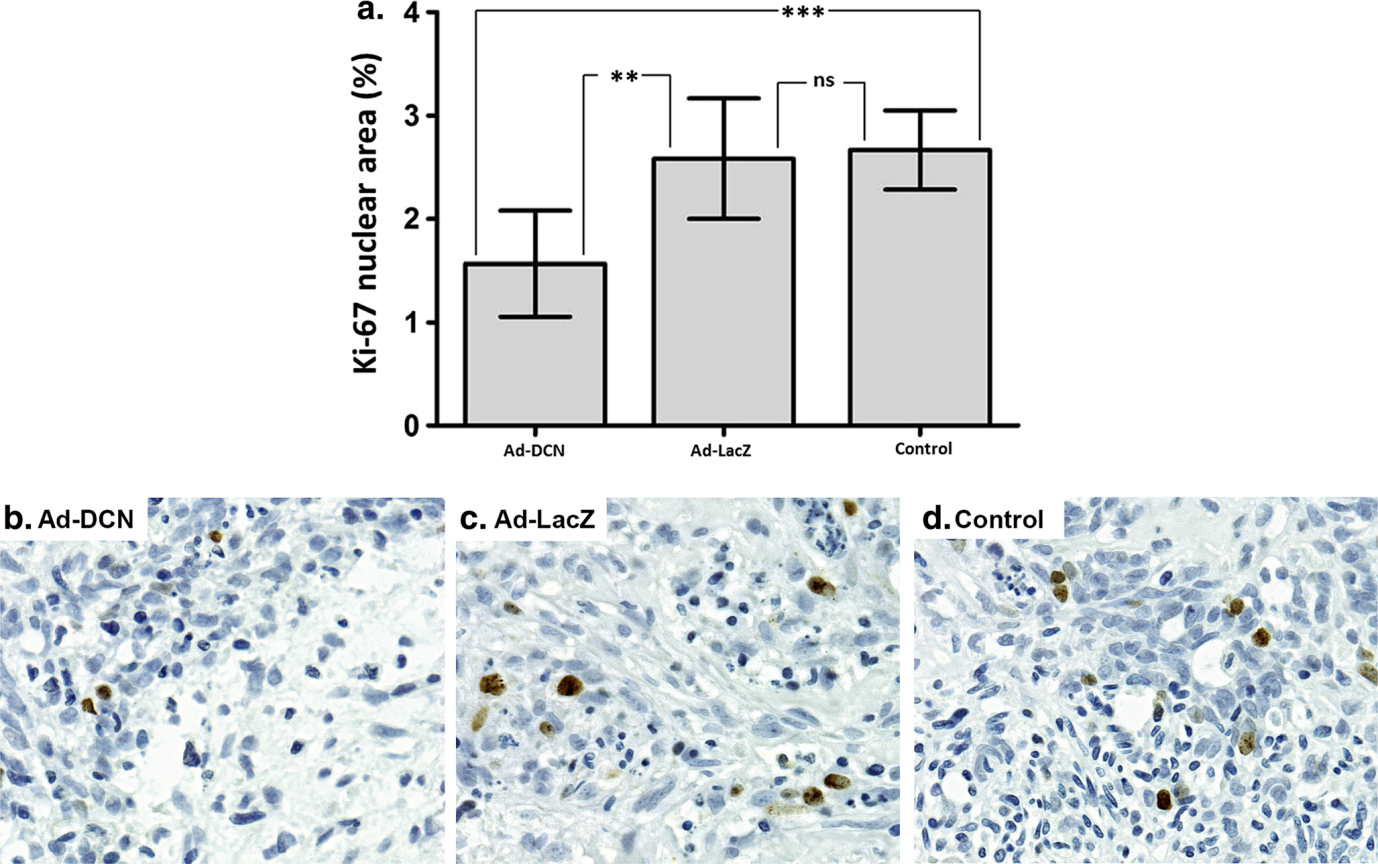
Human decorin cDNA transduction of MBC explant cultures cause a statistically significant decrease in the proliferation index of the cells (using Ki-67). Histogram showing the results obtained from the ImmunoRatio software (**a**). *Capped bars* in the columns indicate SEMs, ** *p* < 0.01; *** *p* < 0.001; ns = non-significant. Representative images of explant cultures transduced with Ad-DCN (**b**) or Ad-LacZ (**c**), and without transduction (control, **d**). Ki-67 positive cells can be seen in *brown*. *Scale bar* 50 μm

Light microscopic observation of 3D MBC explant cultures revealed that Ad-DCN transduction resulted in a marked decrease in the cellularity and in the amount of atypical stromal cells compared to control vector (Ad-LacZ) transduced explants and to negative control cultures (Fig. 5). Explants after Ad-DCN transduction exhibited a lot of fibroblast-like cells in a more organized manner compared to explant cultures transduced with Ad-LacZ or to negative control cultures. Moreover, in Ad-DCN transduced explant cultures, residual rounded cancer cells with hyperchromatic nuclei were distributed in clusters and also areas of cell debris were clearly detected (Fig. 5a). There was also some cell debris present in Ad-LacZ transduced explant cultures. Furthermore, a statistically significant decrease in the proliferation index (using Ki-67 staining) in Ad-DCN transduced explants compared to Ad-LacZ transduced explants or to control explants was observed (Fig. 6).

### Quantitation of mRNA Levels of ErbB Receptors

The expression of the ErbB family members in the transduced BTT samples was studied with RT-qPCR. The results indicated that the MBC tissue expressed roughly similar levels of both EGFR and ErbB2 in all the samples; decorin cDNA and LacZ transduced, and negative controls (Fig. 1, Online Resource 2). No significant expression of ErbB3 or ErbB4 was detected.

## Discussion

In this case report we first demonstrated using multiple immunostainings that the massive breast tumour exhibiting both epithelial and mesenchymal phenotypes was MBC. Next we showed that the tumour was completely negative for decorin immunoreactivity indicating that the cells that formed the MBC tumour were not able to synthesize decorin, a well-known oncosuppressive PG [32, 47]. Instead, there were locally abundant cancer cells that synthesized biglycan, a PG highly similar to decorin but with tumour-promoting function [48–51]. We have previously shown that decorin expression is lacking from Kaposi’s sarcoma and angiosarcoma [52]. Quite recently we have also shown that human ductal, lobular and mucinous breast cancer cells lack decorin synthesis [40]. In all, our previous results together with the present study support the proposal that, mesenchymal and epithelial breast cancer cells are not able to synthesize decorin. Also our yet unpublished studies with testis-derived GTCs support the notion that decorin expression by cancer cells is predominantly absent. However, under certain circumstances some cancer cells, e.g. cancers of blastoma origin [59, 60] or osteosarcomas have been shown to be able to express decorin [61, 62]. Furthermore, proteomic approaches have revealed an association between metastasis and decorin expression in breast cancer [63]. However, it has to be considered that studies based on microarray and proteomic approaches utilizing tumour tissue samples consist not only of cancer cells but additionally of various other cells, e.g. cancer-associated fibroblasts and immune cells, in the TME [61, 63, 64]. Regarding the TME, many cancer types including breast cancer, exhibit phenomena called desmoplasia i.e., the accumulation of ECM macromolecules in the tumour vicinity, which may interfere the interpretation of the methods mentioned above [65–67].

In the present study we established 3D MBC explant cultures to examine whether Ad-DCN transduction might be able to modulate the cytology of the MBC tissue. The results showed that Ad-DCN transduced explant cultures exhibited less cellularity and abundant fibroblast-like cells in a more organized manner compared to explant cultures transduced with Ad-LacZ or to control cultures. Because decorin has been shown to inhibit the epithelial mesenchymal transition (EMT), this increase in fibroblast-like cells is most likely the result of decorin’s capability to normalize the tumour tissue histology rather than to promote more aggressive mesenchymal phenotype typical for MBC [68–70]. Nevertheless, clusters of residual vital cancer cells were detected within areas of cell debris in Ad-DCN transduced cultures. Because some cell debris was also seen in Ad-LacZ transduced cultures this indicates that the Ad-vector by itself has some effect on the cancer cells.

Also the proliferation index of cancer tissue was statistically significantly decreased in response to Ad-DCN transduction. However, some proliferative cells are still seen after Ad-DCN transfection, though adenoviral vectors are known to be able to transduce both non-dividing and dividing cells [71]. Our Ad-vector is a modified Ad5-based non-replicating vector, thus the transfection efficacy is dependent on e.g. adequate virus dosing to the target tumour tissue. Furthermore, the transduction efficacy is subject to the cellular receptors for adenoviruses [72]. For example, coxsackie and adenovirus receptor (CAR) has been identified for Ad5-based vectors, but its expression on different cancer cells can vary and also be downregulated as the cancer progresses [73]. Nevertheless, because the proliferation index is statistically significantly decreased in response to Ad-DCN transduction, it seems that the oncosuppressive effect of decorin cDNA adenoviral transduction applies not only to cancer cells of epithelial origin as we have shown earlier [40, 74, 75], but also to malignant cells exhibiting mesenchymal phenotype. Previously decorin has been shown to be able to suppress also lung metastasis in osteosarcoma [76].

Decorin has also been shown to be able to modulate the signaling pathways of different growth factors such as EGF by binding to its receptors [34, 77]. Particularly the activation of EGFR / ErbB1 and ErbB2 has been shown to be important in the progression of different human cancers, and decorin has been shown to interact with both of them [30, 33, 78, 79]. In this study, the relative expression of ErbB1–4 was examined in transduced BTT samples. The results showed that the MBC tissue expressed both EGFR and ErbB2, but the Ad-DCN transduction was not able to decrease their expression. This is likely due to the vast heterogeneity of the tumour tissue. Furthermore, because the phosphorylation of the ErbB receptors was not examined, the ErbB pathway cannot be definitely excluded from the mechanisms whereby Ad-DCN transduction mediates its oncosuppressive function on MBC cells. In addition to ErbBs, decorin has also been shown to be able to regulate cell-matrix interactions via integrins [80, 81] and to induce apoptosis [82, 83]. Lately, the role of decorin in the regulation of cancer cell mitophagy has gained attention [84]. Furthermore, noteworthy is the idea of “normalization of the tumour microenvironment” whereby decorin in its part could orchestrate cancer cells towards a less malignant phenotype, and maybe even to a less malignant behaviour [85]. Indeed, the importance of decorin as a modifier of the TME in breast cancer has to be recognized [86, 87].

## Conclusions

In this study we have examined decorin in human MBC. We have shown that MBC tissue that is a mixture of epithelial (carcinomatous) and mesenchymal (sarcomatous) elements, is negative for decorin immunoreactivity. The result indicates that the cells of MBC tissue are not able to synthesize this oncosuppressive small PG. We have also shown using 3D MBC explant cultures that decorin cDNA transduction alters the cytological features of the MBC tissue towards a less malignant phenotype. As MBCs form a rare and highly heterogeneous malignancy with poor prognosis due to fairly unsuccessful therapies currently available, novel adjuvant therapeutical approaches are required. The results of the present study favor the use of ECM macromolecules such as decorin in order to orchestrate, reengineer, reprogram, or normalize the structure and composition of the TME to achieve a less friendly environment for the cancer cells.

## Electronic supplementary material

Online Resource 1 (PDF 21 kb)

Online Resource 2 (PDF 19 kb)
