# Supplementary material for: Human Metaplastic Breast Carcinoma and Decorin
Source: Cancer Microenviron. 2017 Jun 26;10(1-3):39–48. doi: 10.1007/s12307-017-0195-8 (PMC5750199; doi:10.1007/s12307-017-0195-8)
Supplement: Supplementary file 1 — (PDF 21 kb) [file 12307_2017_195_MOESM1_ESM.pdf]

Table 1. Primary antibodies used in the IHC stainings. Unless otherwise indicated, antibodies were applied as ready-to-use dilutions and obtained from Ventana, Roche. Antibodies for AR and GCDFP were obtained from Novocastra, Leica Biosystems, and for DCN and BGN from Santa Cruz Biotechnology, Heidelberg, Germany.

**Molecule**	**Clone number**	**Species**
Androgen receptor, AR	AR27, dilution 1:10	Rabbit
Biglycan, BGN	L-15, dilution 1:50	Goat
CD10	SP67	Rabbit
Cytokeratin 5/6, CK5/6	D5/16B4	Mouse
Cytokeratin 7, CK7	SP52	Rabbit
Cytokeratin PAN, CkPan	AE1/AE3&PCK26	Mouse
Decorin, DCN	H-80, dilution 1:50	Rabbit
Epidermal growth factor receptor, EGFR	5B7	Rabbit
Epithelial membrane antigen, EMA	E29	Mouse
Estrogen receptor, ER	SP1	Rabbit
Gross cystic disease fluid protein-15, GCDFP-15	23A3, dilution 1:10	Rabbit
Human epidermal growth factor receptor 2, HER2/neu	4B5	Rabbit
Ki-67	30-9	Rabbit
p63	4A4	Mouse
Progesterone receptor, PR	1E2	Rabbit
Vimentin	V9	Mouse

Human metaplastic breast carcinoma and decorin

Cancer Microenviroment

Pia Boström, Annele Sainio, Natalja Eigėlienė, Anne Jokilammi, Klaus Elenius, Ilkka Koskivuo, Hannu Järveläinen*

*Corresponding author: hanjar@utu.fi,

Department of Medical Biochemistry and Genetics, University of Turku, Kiinamyllynkatu 10, 20520 Turku, Finland and Department of Internal Medicine, Satakunta Central Hospital, Sairaalantie 3, 28500 Pori, Finland
