# Supplementary material for: Human Metaplastic Breast Carcinoma and Decorin
Source: Cancer Microenviron. 2017 Jun 26;10(1-3):39–48. doi: 10.1007/s12307-017-0195-8 (PMC5750199; doi:10.1007/s12307-017-0195-8)
Supplement: Supplementary file 2 — (PDF 19 kb) [file 12307_2017_195_MOESM2_ESM.pdf]

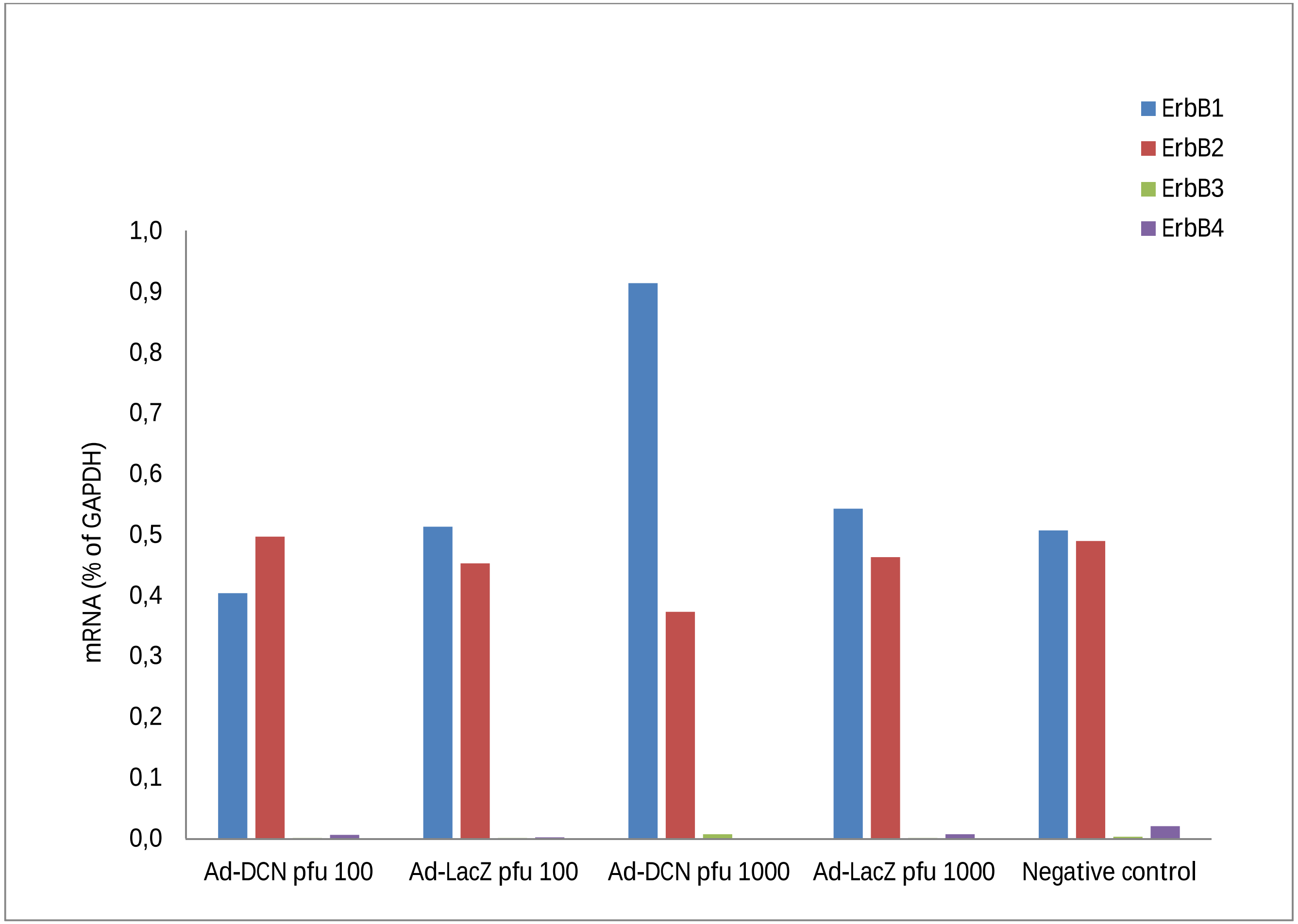


Figure 1. Real-time PCR analysis of ErbB1, ErbB2, ErbB3 and ErbB4 expression in 3D metablastic breast carcinoma (MBC) explant cultures transduced with human adenoviral decorin. ErbB4 expression was determined with primers and probe specific for JM-a isoform. The expressions are relative to the reference gene, GADPH. Ad-DCN, adenoviral vector carrying human decorin cDNA; Ad-LacZ, adenoviral vector control carrying human LacZ gene; and pfu, plaque forming units /cell.

Human metaplastic breast carcinoma and decorin

Cancer Microenviroment

Pia Boström, Annele Sainio, Natalja Eigėlienė, Anne Jokilammi, Klaus Elenius, Ilkka Koskivuo, Hannu Järveläinen*

*Corresponding author: hanjar@utu.fi,

Department of Medical Biochemistry and Genetics, University of Turku, Kiinamyllynkatu 10, 20520 Turku, Finland and Department of Internal Medicine, Satakunta Central Hospital, Sairaalantie 3, 28500 Pori, Finland
